# The Adsorption Effect of Methane Gas Molecules on Monolayer PbSe with and without Vacancy Defects: A First-Principles Study

**DOI:** 10.3390/nano13091566

**Published:** 2023-05-06

**Authors:** Xing Zhou, Yuliang Mao

**Affiliations:** Hunan Key Laboratory for Micro–Nano Energy Materials and Devices, School of Physics and Optoelectronic, Xiangtan University, Xiangtan 411105, China

**Keywords:** methane, monolayer PbSe, first principles, strain

## Abstract

In this paper, the adsorption effect of methane (CH_4_) gas molecular on monolayer PbSe with and without vacancy defects is studied based on first-principles calculations. The effects of the adsorption of methane molecular on monolayer PbSe and on the Se vacancy (V_Se_) and Pb vacancy (V_Pb_) of monolayer PbSe are also explored. Our results show that methane molecules exhibit a good physical adsorption effect on monolayer PbSe with and without vacancy defects. Moreover, our simulations indicate that the adsorption capacity of CH_4_ molecules on monolayer PbSe can be enhanced by applying strain. However, for the monolayer PbSe with Vse, the adsorption capacity of CH_4_ molecules on the strained system decreases sharply. This indicates that applying strain can promote the dissociation of CH_4_ from V_Se_. Our results show that the strain can be used as an effective means to regulate the interaction between the substrate material and the methane gas molecules.

## 1. Introduction

Methane is a colorless, odorless gas that is the main ingredient in natural gas, biogas, etc. It has wide applications for fuel and as a feedstock for synthesizing other substances, such as carbon monoxide and hydrogen. In environmental science, methane has a more significant impact on the Earth’s greenhouse effect than carbon dioxide, and there is a risk of suffocation in environments with high methane concentrations. Therefore, exploring a suitable material for methane gas detection and capture is essential to protect the Earth’s environment and personal safety.

As the first two-dimensional (2D) material discovered, graphene has attracted broad interest due to its unique properties. Two-dimensional materials, due to their structural characteristics, have many potential applications, such as photodetectors [1,2,3,4], field effect transistors [5], solar cells [6,7,8], and gas sensors [9,10,11]. In recent years, more attention has been paid to improving the performance of 2D materials for the applications as gas sensors, such as studies of doping [12,13,14,15], defects [16,17], construction of heterojunctions [18,19], application of external electric fields [20,21] and strain [22,23,24,25]. These methods can effectively tune the interaction between substrate materials and gas molecules.

Among 2D materials, 2D group IV–VI monochalcogenides have attracted much attention due to their unique orthogonal structures, which are like black phosphorus folds. For example, 2D GeSe has attracted significant attention in the study of its application in sensors due to its smooth surface state, good stability, and anisotropic structure [26,27,28]. Recent studies have shown that 2D SnSe has good prospects in near-infrared detectors, high-performance supercapacitors, and solar cells [29,30,31,32,33,34,35]. PbSe, a member of group IV-VI monochalcogenides, was predicted to be used in diodes, infrared lasers, and sensors [36,37,38]. There are currently several methods experimentally used to prepare PbSe. For example, it has been reported that a 2D PbSe semiconductor with large transverse size and ultra-thin thickness was successfully synthesized by van der Waals epitaxy technology [39]. Various lead selenide (PbSe) nanostructures were also prepared using aqueous solutions of Pb(NO_3_)_2_ and NaHSe by varying the molar ratio of Pb and Se and the mixing sequence of NH_4_OH with Pb(NO_3_)_2_ or NaHSe [40,41]. The above methods can produce different shapes of PbSe nanomaterials. A recent study reported that hexagonal PbSe nanostructures can be observed at the interface between gas and liquid, while 2D PbSe superlattices with orthogonal structures can be further synthesized from the formed hexagonal structures [42]. Although different phases of PbSe crystals were prepared experimentally, according to the existing theoretical analysis and research, the hexagonal and orthogonal structures of the monolayer PbSe exhibit good stability at room temperature. Furthermore, it was reported that the orthogonal structure of PbSe is relatively lower in energy than that of the hexagonal PbSe [43,44]. The adsorption of toxic gases (SO_2_ and Cl_2_) on hexagonal PbSe with a single layer, multiple layer, and doped cases was reported [45,46]. In this paper, we mainly choose the relatively more stable orthogonal structure of 2D PbSe as the substrate for the adsorption study. As mentioned above, although there are many related studies on the preparation of PbSe and its application potential in terms of various aspects, the research on gas adsorption of PbSe with a 2D orthogonal structure is still lacking.

In this paper, by using first-principles calculations based on density functional theory (DFT), the adsorption effects of methane gas molecules on 2D PbSe (P-PbSe) with and without vacancy defects (Pb defect: VPb, or Se defect: VSe) are explored. The results show that both P-PbSe and PbSe with Se atomic vacancy (VSe) exhibit significant adsorption effects on CH4. In addition, strain control is a valid method for regulating two-dimensional materials, and there are a large number of related studies on the theoretical calculations of gas adsorption on two-dimensional materials [47,48,49]. Some studies have reported the effect of strain on the electronic and optical properties of PbSe with a 2D orthogonal structure. In this paper, we mainly aim to explore the adsorption of methane molecules on 2D PbSe after the biaxial strain is applied. To ensure that the structure of the calculated material is not distorted under biaxial strain, only the strain in a range from −5% to 5% is considered in our study (where a negative value indicates compressive strain and a positive value indicates tensile strain). In previous reports on gas adsorption research, for substrate materials, only the structure subject to defects or the pristine substrate structure under strain was considered to explore the changes in their adsorption performance. However, research on the application of external strain to the structure under defect conditions in gas adsorption has not yet been explored. Therefore, in this article, the adsorption of methane molecules on the optimal sites of P-PbSe and VSe after applying biaxial strain is systematically studied.

## 2. Computational Method and Model

The first-principles calculations are performed based on DFT. The geometric structure and electronic properties of the system are simulated using the 5.44 version of Vienna ab initio simulation package (VASP.5.4.4) [50,51]. The exchange-correlation interaction between electrons was described by the Perdew–Burke–Ernzerhof (PBE) function in Generalized Gradient Approximation (GGA) [52,53]. The cutoff energy for the expanding plane wave function was set to 500 eV. The convergence criterion in energy and force was set to 10^−6^ eV and 0.01 eV/Å, respectively. In the integration of the Brillouin zone (BZ), the 7 × 7 × 1 K-grid mesh was used. The DFT-D3 method was applied to describe the van der Waals forces between substrate materials and gas molecules [54,55]. A vacuum layer of 25 Å was set along the z-axis to ensure there is no interaction between the adjacent layers. Strain can affect the bonding between atoms in the material. When strain is applied externally, the lattice constants are changed due to the rearrangement of atoms in the material, and the atomic spacing of the crystal is also changed. Under compressive strain, the lattice constant will decrease. Thus, the distance between the atoms is reduced, and the atoms are arranged more closely. In contrast, under tensile strain, the lattice constant will be increased. Correspondingly, the distance between atoms will become larger, which leads to the weakening of the interaction force between atoms and bonds. In this case, the crystal structure becomes looser. Considering the rationality of the configurations, we calculated the molecular dynamics of the systems under different strains. Ab initio molecular dynamics (AIMD) simulations were performed to verify the thermodynamic stability of the relaxed configurations. The NVT ensemble was chosen in AIMD simulations under 300 K. A 3 × 1 × 1 supercell was used to predict the thermodynamic stability of the system under different strains. The optimized adsorption energy (Ead) of CH_4_ molecular adsorbed on the PbSe substrate is defined as:

$$E_{\mathrm{ad}}=E_{\mathrm{tol}}-E_{p}-E_{\mathrm{gas}}$$

where Etol represents the total energy of the adsorbed configurations, and E_p_ and E_gas_ represent the total energy of the PbSe monolayer and the gas molecules, respectively.

The biaxial strain was applied to the PbSe substrate, and the strain variability is defined as:

$$\frac{n-n_{0}}{n_{0}}\;\times \;100\%$$

where *n* and $n_{0}$ are the lattice constants of the system with and without strain, respectively.

## 3. Results and Discussion

### 3.1. Adsorption of Methane Molecules on Pristine 2D PbSe

**Figure 1:**
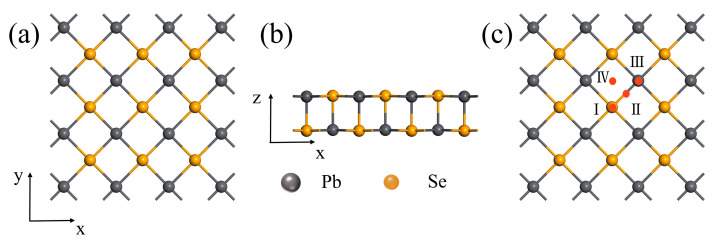
(**a**,**b**) represent the top and side views of the single-layer PbSe, respectively. (**c**) represents four different adsorption sites of P-PbSe (Ⅰ: TSe site, Ⅱ: Bridge site, Ⅲ: TPb site, Ⅳ: Hollow site); the gray and green balls represent Pb and Se atoms, respectively.

In order to ensure the rationality of the calculated structures, the structure of the unit cell of PbSe is firstly optimized. In unit cell, the optimized lattice constants are a = b = 4.40 Å. A 3 × 3 × 1 supercell containing 18 Pb atoms and 18 Se atoms was used as the adsorbent substrate material in our lateral calculations with the optimized lattice constant a = b = 13.205 Å. The band structure and density of states (DOS) of the optimized system were calculated. The results show that PbSe is a direct bandgap semiconductor with a bandgap of 1.28 eV, which is consistent with previous reports [56,57]. Methane gas molecules have a regular tetrahedral structure, so only one configuration is considered in the study of the adsorption configuration, i.e., the adsorption configuration of the bonding between the C atom and the H atom in the methane molecule is perpendicular to the PbSe surface. The top view and side view of the optimized structure of PbSe after cell expansion are shown in Figure 1a and Figure 1b, respectively. Four different adsorption sites are proposed, which are named Ⅰ (the top site of the Se atom, named the TSe site), Ⅱ (located directly above the bonding between the Pb atom and Se atom, named the Bridge site), Ⅲ (the top site of the Pb atom, named the T_Pb_ site), and Ⅳ (hollow site) corresponding to the different high symmetry adsorption sites of methane molecules on the P-PbSe substrate, as shown in Figure 1c.

In order to obtain the optimal adsorption site of methane molecules adsorption on 2D PbSe, the adsorption energies of methane molecules on four different sites of P-PbSe were calculated, and the results are listed in Table 1. Based on the above-mentioned formula of adsorption energy, the larger the absolute value of the adsorption energy E_ad_, the stronger the adsorption capacity. By comparing the E_ad_ listed in Table 1, the proposed site for methane molecule adsorption on P-PbSe is the T_Pb_ site. As can be found from the data in Table 1, the difference in the adsorption energies of methane molecules on the four sites of P-PbSe or V_Se_ is not very obvious, which means that methane molecules have almost the same adsorption stability on different sites. Therefore, only the adsorption sites with the lowest adsorption energy are considered for further analysis in the subsequent discussion.

**Figure 2:**
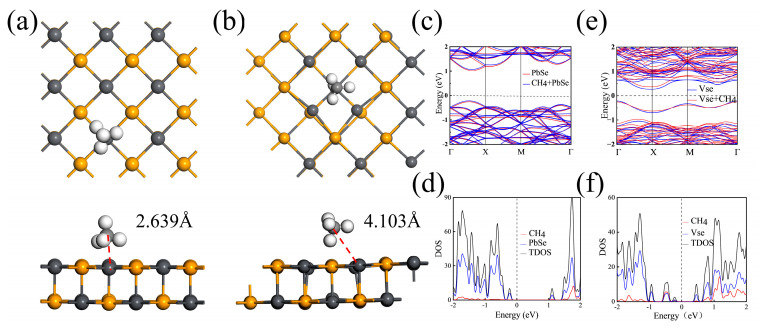
(**a**,**b**) are the configurations of CH_4_ molecules adsorbed on the sites of P-PbSe and V_Se_, respectively. (**c**,**d**) and (**e**,**f**) are the corresponding band structure and electronic density of states (DOS), respectively.

Therefore, the T_Pb_ site is mainly considered for the adsorption of methane molecules on 2D P-PbSe substrate. The top view and side view of the relaxed structure of methane molecules adsorbed on the T_Pb_ site are shown in Figure 2a. The distance from the carbon atom in the methane molecule to the nearest atom (Pb) on the substrate plane is 2.639 Å. The band structure with and without the adsorption of methane molecules on P-PbSe substrate is shown in Figure 2c, and the electronic density of states after adsorption is shown in Figure 2d. The charge of each atom of a single methane molecule and methane molecules adsorbed on T_Pb_ sites, and the electron transfer of each atom in methane molecules after adsorption, are listed in Table 2. As shown in Figure 2c, the variation in the band gap in the configurations with and without methane molecules is not remarkable. In Figure 2d, it can be seen that methane molecules do not obviously contribute to the states of valence and conduction bands. Combined with the charge transfer in Table 2, it can be concluded that physical adsorption of methane molecules occurs on P-PbSe, which mainly depends on the van der Waals (vdW) interaction between the substrate and methane molecules.

### 3.2. Adsorption of Methane Molecules on V_Se_

**Figure 3:**
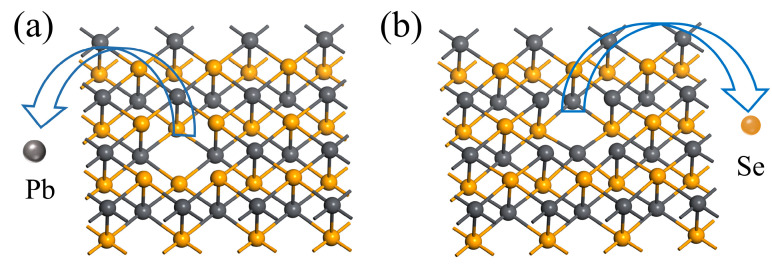
(**a**,**b**) denote vacancy configurations of Pb atoms and Se atoms, respectively.

When considering the vacancy in monolayer PbSe, two cases are considered. One is Pb atoms vacancy (V_Pb_) and the other is Se atoms vacancy (V_Se_). Their configurations are shown in Figure 3a and Figure 3b, respectively.

To gain a further understanding of the two vacancy cases, the energies of the two vacancy configurations are calculated separately. The results show that the energy of the V_Se_ configuration (−139.49 eV) is lower than that of the V_Pb_ configuration (−139.01 eV). From the perspective of structural stability, a more stable V_Se_ configuration is considered for the lateral discussion in this paper.

**Figure 4:**
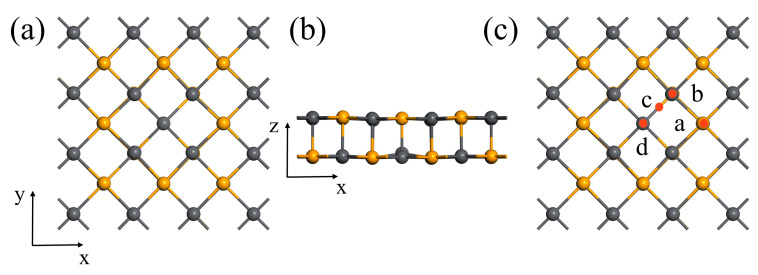
(**a**,**b**) represent the top and side views of optimized Vse configuration. (**c**) Four different sites of Se atomic vacancy (a: T_Se_ site, b: Bridge site, c: T_Pb_ site, d: Hollow site).

The top and side views of the V_Se_ optimized configurations are shown in Figure 4a and Figure 4b, respectively. We also consider four different adsorption sites. As shown in Figure 4c, a is the top site of the Pb atom (T_Pb_), while b is the Bridge site between the Pb atom and Se atom (Bridge). c is the top site of the Se atom (T_Se_), while h is the site above the Se atom vacancy (Hollow). The adsorption energies of the four sites were calculated, and the results are listed in Table 1. By comparison of the adsorption energies, it is found that the optimal adsorption site of CH_4_ on V_Se_ is the Hollow site, and the adsorption energy is −4.6885 eV. It should be noted that the adsorption energies between the four adsorption sites in the V_Se_ system have only minor differences. Usually, only the site with lowest adsorption energy among them is selected as a representative for further calculation and analysis. The top view and side view of the optimized structure are shown in Figure 2b. From our analysis, the distance from the carbon atom in the methane molecule to the nearest atom (Pb) on the substrate plane is 4.103 Å.

In order to further explore the charge transfer of the configurations after adsorption, Bader charge analysis was carried out to analyze the charge of each atom of a single methane molecule adsorbed on the Hollow site of the V_Se_, and the electron transfer of each atom in methane molecules after adsorption is listed in Table 3. It can be seen that only 0.03 e is transferred from the V_Se_ configuration to CH_4_ molecules after adsorption. Combined with the band structure in Figure 2e and the electronic density of states in Figure 2f, it can be concluded that the adsorption of a single methane molecule has a minor influence on the band gap and electronic state of the system, which indicates that the adsorption type between V_Se_ and methane molecules is physical adsorption.

### 3.3. Adsorbtion Methane Molecules on the PbSe Monolayer under Biaxial Strain

**Figure 5:**
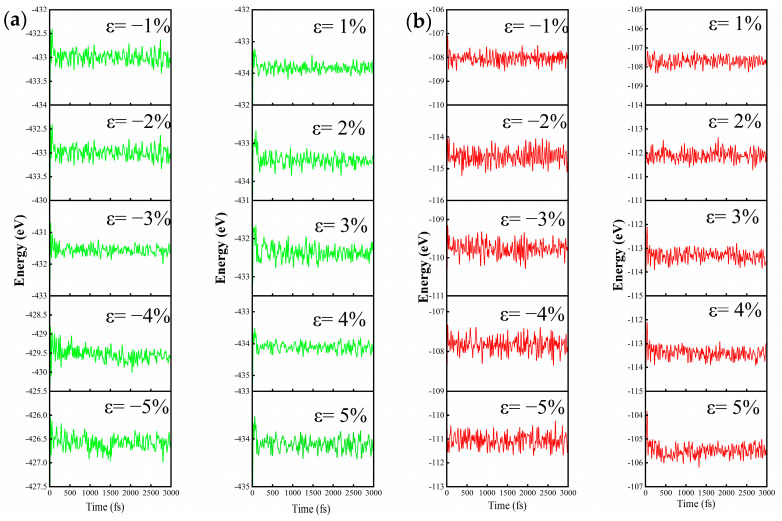
The free energy fluctuations of P-PbSe (**a**) and V_Se_ (**b**) at room temperature after molecular dynamics simulation under different strains.

When strain is applied to P-PbSe and V_Se_ configurations, the thermodynamic stability of the structure under strain is checked first to ensure the rationality of the explored structure. The variation in free energies in the studied configurations of P-PbSe and V_Se_ under applied strain from −5% compressive strain to 5% tensile strain are shown in Figure 5. It can be seen that the free energies of P-PbSe and V_Se_ only fluctuate in a minimal interval under different strains, which indicates that the structure is stable from −5% compressive strain to 5% tensile strain.

**Figure 6:**
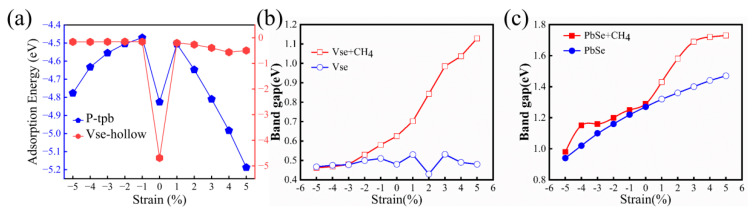
(**a**) The adsorption energy of P-PbSe (blue part) and V_Se_ (red part) for methane molecules under different strains; (**b**,**c**) show the change in the band gap of V_Se_ and P-PbSe with and without adsorbed methane molecules as a function of the response variable strain, respectively.

In order to explore the effect of strain applied to the substrate material on the adsorption energy of gas molecules, the adsorption energy of CH_4_ molecules on P-PbSe and V_Se_ under different strains is indicated in Figure 6a. It is shown that the strain has an effective control effect on the adsorption capacity of the substrate material. It can be found that when the strain is applied, the adsorption energy of P-PbSe is decreased, but as the strain gradually increases, the adsorption energy of P-PbSe is increased with the increase of the biaxial strain. When the tensile strain of 4% is applied, the adsorption capacity of CH_4_ molecules is significantly improved when compared with the case without strain. This implies that the adsorption capacity of CH_4_ molecules on P-PbSe can be enhanced by applying strain, which is beneficial to its application in the capture of CH_4_ molecules. However, for the V_Se_ system, the adsorption capacity of CH_4_ molecules on the strained system decreases sharply. This indicates that applying strain can promote the dissociation of CH_4_ from V_Se_. In other words, the strain can be used as an effective means to regulate the interaction between the substrate material and the methane gas molecules.

To further explore the effect of strain on the electronic structure of the adsorbed system, we calculated the band structure of the configurations with and without adsorption of CH_4_ molecules on P-PbSe and V_Se_ configurations under different strain. The results are shown in Figure 6b and Figure 6c, respectively. It can be seen from Figure 6b that strain does not change the band type of the V_Se_ configuration. However, for the system with adsorbed methane molecules, the band gap is gradually increased with the increase of strain from −5% to 5%. It can be seen that the change in the band gap of the system with and without adsorption under compressive strain is not significant. Especially at strains ranging from −5% to −3%, the effect of strain on its band gap is almost consistent. However, at strains ranging from −2% to 5%, the band gap of the adsorbed system gradually increases, while for the V_Se_ system, the band gap fluctuates within a certain range. However, the band gap of methane molecule adsorption on P-PbSe increases under the applied strain from −5% to 5%. Furthermore, the type of the band structure in these cases changes from direct to indirect during the change from compressive to tensile strain. Although the band gap varies under the strain ranging from −5% to 5%, the types of band gap in the P-PbSe system with and without adsorbing methane molecules are unchanged. Unlike V_Se_, there is a significant difference in the band gap between the system with and without adsorption at strains ranging from −5% to −3%, while at strains ranging from −2% to −1%, the band gap distinction is negligible. However, there is a significant discrepancy in the response of the band gap to strain between the two under 1% to 5% strain. For the V_Se_ configuration and P-PbSe, the band gap of the system after the adsorption of methane molecules has a sensitive response to the strain, and the band gap of the system is changed more significantly, especially under tensile strain.

**Figure 7:**
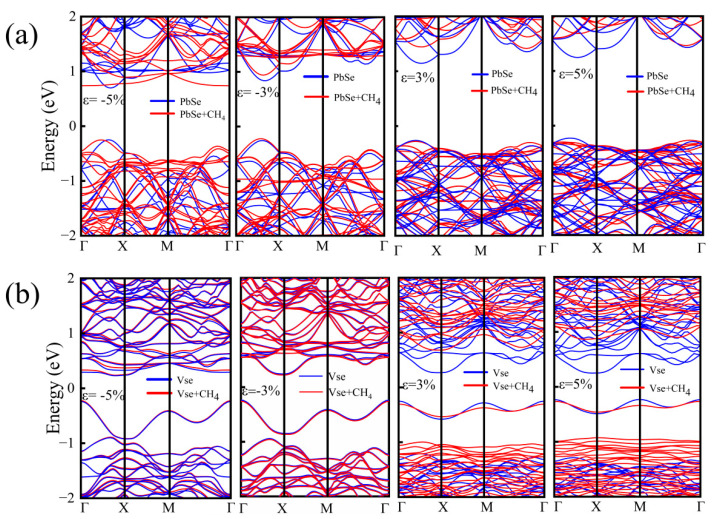
(**a**,**b**) are the band structures of P-PbSe and V_Se_ configurations with and without adsorption of methane molecules under 3% and 5% compressive and tensile strains, respectively.

The band structures of the systems with and without the adsorption of CH_4_ molecules on P-PbSe and V_Se_ configurations under selected strains are shown in Figure 7. It can be seen from the figure that, whether in the P-PbSe or V_Se_ system, the compressive strain has no noticeable effect on the band structure of the system adsorption of methane molecules. On the contrary, the tensile strain significantly changes the electronic states in the conduction band minimum (CBM) of the adsorbed system, especially for the adsorption of methane molecules on the V_Se_ system.

The results of the change in adsorption energy, the change in band gap, and the charge transfer between the substrate material and methane molecules of P-PbSe and V_Se_ systems under different strains are listed in Table 4 and Table 5, respectively. From the data in the table, it can be seen that strain has a significant regulatory effect on the band gap and adsorption energy of the systems after adsorption of CH_4_ molecules, but its role in charge transfer is very limited.

## 4. Conclusions

In this paper, based on first-principles calculations, we calculate the adsorption energy, band structure, and Bader charge of CH_4_ molecules’ adsorption on P-PbSe and V_Se_ substrate materials. The results show that CH_4_ molecules exhibit physical adsorption effects on both P-PbSe and V_Se_ substrate. The effect of biaxial strain on the adsorption of CH_4_ molecules on P-PbSe and V_Se_ was calculated. Our analysis of band structure reveals that the systems with and without adsorption of CH_4_ molecules have a prominent difference. The changes in the band gap of the adsorbed system are more sensitive to strain. However, the effect of strain on charge transfer is very limited. Our study indicates that strain can be used as an effective method to regulate the electronic structure of methane molecules adsorbed on 2D PbSe nanomaterial. Comparing the calculation results of P-PbSe and V_Se_ under strain, it is found that V_Se_ and P-PbSe have significantly different responses to methane molecule adsorption under the same strain. Therefore, applying strain to a substrate material such as 2D PbSe is an effective way to regulate the gas adsorption performance.

## Figures and Tables

**Table 1:** Adsorption energies of methane molecule adsorption on P-PbSe and PbSe with V_Se_.

Site	T_Pb_	T_Se_	Bridge	Hollow
P-PbSe (eV)	−4.8259	−4.7887	−4.8158	−4.8177
V_Se_-PbSe (eV)	−4.6776	−4.6506	−4.6467	−4.6885

**Table 2:** The Bader charge of each atom in a single methane molecule (CH_4_) and methane molecules adsorbed on TPb sites (CH_4_-T_Pb_). The charge transfer of each atom of methane molecules is also listed (positive indicates electron gain, negative indicates electron loss).

Atom	CH_4_ (e)	CH_4_-T_Pb_ (e)	Transfer (e)
C	4.09581	4.115225	+0.019415
H1	0.967049	0.985145	+0.018096
H2	0.967049	0.955066	−0.011983
H3	0.992434	0.988498	−0.003936
H4	0.977659	0.967289	−0.01037

**Table 3:** The charge of each atom of a single methane molecule (CH_4_) and methane molecules adsorbed on the Hollow site of the vacancy of the Se atom (CH_4_-Hollow). The charge transfer of each atom of the methane molecule (positive indicates electron gain, negative indicates electron loss).

Atom	CH_4_ (e)	CH_4_-Hollow (e)	Transfer (e)
C	4.09581	4.110206	+0.014396
H1	0.967049	0.980938	+0.013889
H2	0.967049	0.979328	+0.012279
H3	0.992434	0.961884	−0.03055
H4	0.977659	0.999377	+0.021718

**Table 4:** Variation in adsorption energy (E_ad_), band gap (E_gap_), and charge transfer after adsorption of methane molecules on P-PbSe at different strains. Positive charge transfer means the methane molecule gained electrons.

Strain (%)	E_ad_ (eV)	E_gap_ (eV)	Transfer (e)
−5	−4.767	0.99	0.0175
−4	−4.624	1.15	0.0131
−3	−4.555	1.16	0.0123
−2	−4.504	1.21	0.0107
−1	−4.471	1.26	0.0104
0	−4.826	1.29	0.0112
1	−4.505	1.43	0.0118
2	−4.647	1.58	0.0113
3	−4.81	1.7	0.0108
4	−4.984	1.72	0.0108
5	−5.187	1.73	0.0104

**Table 5:** Variation in adsorption energy (E_ad_), band gap (E_gap_), and charge transfer amount after adsorption of methane molecules on V_Se_ under different strains. Positive charge transfer means the methane molecule gained electrons, respectively.

Strain (%)	E_ad_ (eV)	E_gap_ (eV)	Transfer (e)
−5	−0.161	0.46	0.028
−4	−0.161	0.47	0.028
−3	−0.157	0.48	0.029
−2	−0.15	0.53	0.031
−1	−0.155	0.58	0.031
0	−4.689	0.63	0.03
1	−0.202	0.7	0.03
2	−0.265	0.84	0.029
3	−0.394	0.99	0.029
4	−0.563	1.04	0.228
5	−0.498	1.13	0.027

## Data Availability

The data used in this study are available from the corresponding author by request.
